# Fluorescent image-guided surgery in breast cancer by intravenous application of a quenched fluorescence activity-based probe for cysteine cathepsins in a syngeneic mouse model

**DOI:** 10.1186/s13550-020-00688-0

**Published:** 2020-09-29

**Authors:** Frans V. Suurs, Si-Qi Qiu, Joshua J. Yim, Carolien P. Schröder, Hetty Timmer-Bosscha, Eric S. Bensen, John T. Santini, Elisabeth G. E. de Vries, Matthew Bogyo, Gooitzen M. van Dam

**Affiliations:** 1 Department of Medical Oncology, University of Groningen, University Medical Center Groningen, Groningen, The Netherlands; 2 Diagnosis and Treatment Center of Breast Diseases, Affiliated Shantou Hospital, Sun Yat-Sen University, Shantou, China; 3 Department of Chemical and Systems Biology, Stanford University School of Medicine, Stanford, CA USA; 4 Vergent Bioscience, Inc., Minneapolis, MN USA; 5 Department of Pathology, Stanford University School of Medicine, Stanford, CA USA; 6 Department of Microbiology and Immunology, Stanford University School of Medicine, Stanford, CA USA; 7 Department of Surgery, University of Groningen, University Medical Center Groningen, Groningen, The Netherlands; 8 Department of Nuclear Medicine and Molecular Imaging and Medical Imaging Center, University of Groningen, University Medical Center Groningen, Groningen, The Netherlands

**Keywords:** Image-guided surgery (IGS), Quenched fluorescent activity-based probe (qABP), Cathepsin targeting, Indocyanine green (ICG), Breast cancer

## Abstract

**Purpose:**

The reoperation rate for breast-conserving surgery is as high as 15–30% due to residual tumor in the surgical cavity after surgery. In vivo tumor-targeted optical molecular imaging may serve as a red-flag technique to improve intraoperative surgical margin assessment and to reduce reoperation rates. Cysteine cathepsins are overexpressed in most solid tumor types, including breast cancer. We developed a cathepsin-targeted, quenched fluorescent activity-based probe, VGT-309, and evaluated whether it could be used for tumor detection and image-guided surgery in syngeneic tumor-bearing mice.

**Methods:**

Binding specificity of the developed probe was evaluated in vitro. Next, fluorescent imaging in BALB/c mice bearing a murine breast tumor was performed at different time points after VGT-309 administration. Biodistribution of VGT-309 after 24 h in tumor-bearing mice was compared to control mice. Image-guided surgery was performed at multiple time points tumors with different clinical fluorescent camera systems and followed by ex vivo analysis.

**Results:**

The probe was specifically activated by cathepsins X, B/L, and S. Fluorescent imaging revealed an increased tumor-to-background contrast over time up to 15.1 24 h post probe injection. In addition, VGT-309 delineated tumor tissue during image-guided surgery with different optical fluorescent imaging camera systems.

**Conclusion:**

These results indicate that optical fluorescent molecular imaging using the cathepsin-targeted probe, VGT-309, may improve intraoperative tumor detection, which could translate to more complete tumor resection when coupled with commercially available surgical tools and techniques.

## Introduction

Breast cancer is the most commonly diagnosed cancer and the leading cause of cancer-related mortality in females worldwide [1]. Surgical resection is the standard of care primary treatment modality for patients with early breast cancer. Combined with radiotherapy, breast-conserving surgery demonstrates comparable patient outcome with mastectomy, while preventing unnecessary excision of normal breast tissue [2, 3]. Local recurrence is mainly due to residual tumor in the surgical cavity. The risk of local recurrence is reduced by reoperation if a tumor-positive margin is found by histopathological examination [4]. An important downside of the use of breast-conserving surgery is the high reoperation rate after the initial lumpectomy, ranging from 15 to 30% [5, 6]. Current methods for intraoperative margin assessment in breast-conserving surgery include gross pathologic examination, frozen section analysis, imprint cytology, and radiofrequency spectroscopy (MarginProbe). While these techniques have been shown to reduce high reoperation rates during breast-conserving surgery, there is insufficient evidence that they are effective when baseline rates are below 20% [7]. Drawbacks to these methods include sampling bias and error due to the small amount of tissue analyzed during frozen section and imprint cytology analysis, the need for an experienced cytopathologist for accurate imprint cytology interpretation, and high false-positive rates observed with the MarginProbe device [8]. Therefore, there is an unmet need for new techniques to improve positive margin detection intraoperatively and consequently reduce reoperation rates. Tumor-targeted optical molecular imaging provides an opportunity to identify malignant lesions in the tumor bed and could potentially meet this need. Particularly, probes activated by tumor-specific enzymes offer high tumor-to-background contrast because inactivated agents yield little signal [9, 10].

Cysteine cathepsin proteases are a class of highly evolutionarily conserved lysosomal enzymes that play an important role in neoplastic transformation [11]. The secreted cathepsins degrade extracellular matrix and cleave cell-cell adhesion molecules, which promote tumor cell invasion and metastasis [12, 13]. In human cancers, including breast cancer, cathepsin expression is frequently increased compared with normal tissue [14].

In breast cancer, cathepsins B, L, C, and S are highly expressed in the tumor tissue and are associated with poor patient outcomes [11]. Cathepsins can be expressed on cancer cells as well as immune cells such as macrophages [11, 13]. This data provided the rationale for targeting cathepsins with a molecular imaging agent to improve intraoperative tumor margin assessment. We evaluated an activity-based approach. This method provides low-background signal and high-contrast fluorescent images after activation of the probe. Upon covalent and irreversible binding to the active site of an enzyme, a fluorescently quenched activity-based probe (qABP) is activated and emits the fluorescent signal [15]. BMV109, a qABP probe containing a Cy5 fluorophore for tumor detection covalently binds to active cysteine cathepsins [16]. It has demonstrated in vivo tumor labeling when administered by intravenous injection in murine breast and colon cancer models as well as labeling of the human breast, lung, liver, pancreas, and colon cancer tissues when applied topically [9, 16, 17].

**Fig. 1:**
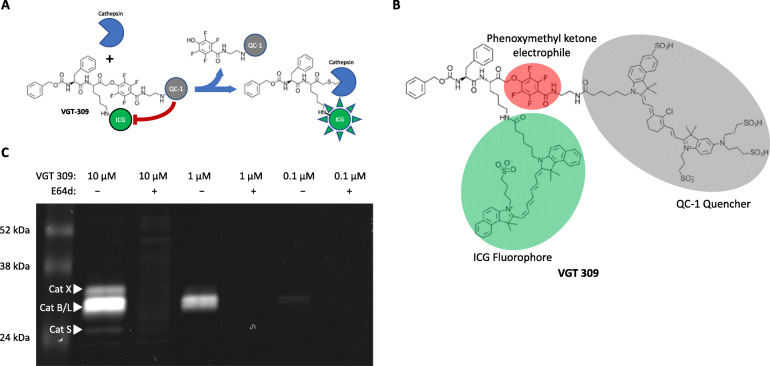
Mode of action and structure of VGT-309 and in vitro binding to active cysteine cathepsins. **a** Mode of action of VGT-309. Cathepsins covalently and irreversibly bind VGT-309, displacing the quencher QC-1. With the quencher displaced, the ICG fluorescence of VGT-309 is no longer quenched and the probe becomes fluorescent. **b** Structure of VGT-309. The phenoxymethyl ketone electrophile (red) forms a covalent bond with the active cysteine of cathepsins. The fluorophore ICG (green) is for visualization and the quencher (gray) prevents fluorescent signal in the free, unbound probe. **c** VGT-309 labeling of endogenously expressed cathepsins in RAW-264.7 cells. Cells were treated with probe for 30 min in the presence or absence of cysteine cathepsin inhibitor E64d followed by lysis and analysis by SDS-PAGE and scanning for fluorescent labeled proteins. The locations of cathepsin X, B/L, and S are indicated

To ensure compatibility with commercially available, near-infrared surgical imaging systems and facilitate rapid clinical translation, a new qABP, VGT-309, was developed by replacing the Cy5 fluorophore in BMV109 with indocyanine green (ICG) and the sulfo-QSY21 quencher with QC-1 (Fig. 1a and Fig. S1). ICG has longer excitation and emission wavelengths and is approved by the US Food and Drug Administration (FDA) and European Medicines Agency (EMA) for human use. In this proof of concept study, we evaluated VGT-309 for in vivo tumor detection and guiding surgical tumor excision using different clinical imaging devices in a 4T1 mammary tumor allograft model. In addition, we investigated which cellular sources in the tumor would contribute to the signal of VGT-309.

## Materials and methods

### Probe and cell lines

qABP VGT-309 was provided by Vergent Biosciences, Minnesota. The synthesis and evaluation of the related qABP BMV109, which selectively targets a broad spectrum of cathepsins, has been previously reported [16]. The probe used in this study, VGT-309, uses the same cathepsin recognition sequence and phenoxymethyl ketone as BMV109, but here the far-red Cy5 fluorophore is replaced with the near-infrared ICG (Intrace Medical) and the sulfo-QSY21 quencher (Life Technologies Corporation) with QC-1 (LI-COR Biosciences) (Fig. 1a and Supplementary Fig. S1).

The murine breast cancer cell line 4T1 (American Type Culture Collection) was cultured in RPMI-1640 medium (Invitrogen) containing 10% fetal calf serum (Bodinco BV). The murine macrophage cell line RAW 264.7 (American Type Culture Collection) was cultured in Dulbecco’s modified eagle medium (Gibco) containing 10% fetal calf serum.

Cells were used between passages 5–30 after thawing to ensure complete revival and were routinely tested for the presence of mycoplasma. Cells were cultured under aseptic conditions at 37 °C in an incubator providing humidified atmosphere of 5% CO_2_ in air.

### In vitro VGT-309 activation

RAW 264.7 cells were harvested, counted, and diluted to 2 × 10^6^ cells/mL in phosphate-buffered saline (PBS). The cells were incubated in the presence or absence of 500 μM of cathepsin inhibitor E64d (Santa Cruz Biotechnology) for 30 min at 37 °C followed by the addition of VGT-309 at the indicated concentrations for 30 min at 37 °C. The cells were spun down at 2287 rcf, the remaining inhibitor/VGT-309 solution was removed. The cells were washed twice with PBS by spinning down and removing the supernatant. The cells were resuspended in PBS and put through two freeze-thaw cycles (snap freeze with liquid nitrogen/40 °C). To each tube, 4x SDS-sample buffer containing 2-mercaptoethanol was added followed by heating for 3 min at 100 °C. Proteins were resolved by SDS-PAGE (12%) and the gel was scanned with an Odyssey CLx Imaging System (LI-COR Biosciences) on the 800 nm channel to visualize VGT-309-labeled proteases. Images were analyzed using Image Studio (LI-COR Biosciences) software.

### Animal experiments

Animal experiments were approved by the Institutional Animal Care and Use Committee of the University of Groningen and performed according to Dutch law. Eight- to 10-week-old female BALB/c mice (BALB/cOlaHsd, Envigo) were allowed to acclimatize for 1 week. The mice received an alfalfa-free diet. The mice were injected with 5 × 10^4^ 4T1 cells in 50 μL RPMI-1640 in the lower mammary fat pad. After 19–21 days, the imaging experiments were performed when the tumors reached ± 400 mm^3^. Mice without tumor implantation were used as a negative control model. The mice were intravenously injected with 100 μL of 0.5 mg/mL VGT-309 (20 nmol). Mice were anesthetized with isoflurane/medical air inhalation (5% induction, 2.5% maintenance).

### In vivo fluorescent imaging

Tumor-bearing mice were imaged with the IVIS Spectrum (PerkinElmer) at 1, 2, 4, 8, and 24 h after injection. The control group was imaged 24 h after injection. Six mice were evaluated at each time point. Settings used for the IVIS Spectrum were medium binning and FStop 1 with 2 s exposure. Excitation was at 745 nm with emission at 820 nm. Before imaging, hair was removed via shaving and with hair removal lotion. After in vivo imaging, mice were sacrificed via cervical dislocation while under anesthesia.

### Ex vivo fluorescent imaging

Tissues of interest were collected, namely the tumor, liver, lung, heart, spleen, kidney, brain, fat, skin, muscle, and pancreas. They were placed on wet gauzes and imaged with the Pearl imager (Li-COR Biosciences) before formalin-fixation in 4% paraformaldehyde (PFA)/PBS and paraffin embedding. The Pearl imager has an excitation laser of 785 nm and detects all fluorescence above 820 nm. For analysis of the images taken with the Pearl, regions of interests (ROIs) were drawn around the tissues in the white-light images and these ROIs were subsequently quantified on the fluorescent images with ImageJ.

### Image-guided surgery

Two mice per time point were scanned with the IVIS Spectrum. Time points were 1, 2, 4, 8, and 24 h after injection. After the mice were sacrificed via cervical dislocation the tumor was surgically removed while using an image-guided surgery system. At each time point, the surgical procedure was recorded for two mice, one mouse with the Explorer Air (SurgVision) and one mouse with the Spy Elite (Novadaq). The working distance was 20 cm above the surgical field. The upper part of the mice was covered to prevent influence from the scattered fluorescence of the liver.

The Explorer Air has two LED lights for 800 nm illumination and one LED light for white light illumination, enabling both fluorescence and white light images to be simultaneously recorded. The Spy Elite could only record the fluorescent image. Therefore, when performing image-guided surgery using the Spy Elite, the Explorer air system was used to record the white light image. After surgery, the tumor and the tissues of interest were collected, formalin-fixed for 6 h in 4% PFA/PBS, followed by overnight storage in 30% sucrose/PBS and frozen with TissueTek O.C.T compound (Sakura).

### Ex vivo tissue analysis

To enable further ex vivo analysis, tumor, muscle, and spleen tissues of mice 24 h after probe injection were sliced into 4-μm sections and, where needed, deparaffinized for scanning with the Odyssey CLx flatbed scanning system with the following settings: wavelength = 800 nm, resolution = 21 μm, quality = high, intensity = 3. Next, these slides were stained with hematoxylin and eosin (H&E). Macrophages were visualized with immunohistochemical (IHC) staining for murine F4/80 with a rat anti-mouse F4/80 antibody, clone: CI:A3 (Bio-Rad laboratories). After antigen retrieval for 15 min at 95 °C with a citrate buffer (pH 6), slides were incubated with a primary antibody with a dilution of 1:250 overnight. Next, a rabbit anti-rat antibody (Dako) followed by a peroxidase conjugated goat anti-rabbit antibody (Dako) was used. Finally, 3-3′-diaminobenzidine was added to visualize peroxidase activity. Slides were evaluated by a pathologist.

### Statistical analysis

All data are presented as mean ± standard error. An independent Student’s *T* test was performed to test differences between groups (GraphPad, Prism 7). A *P* value of less than 0.05 was considered significant.

## Results

### qABP VGT-309 activation is inhibited in vitro by a cathepsin activity inhibitor

The binding specificity of VGT-309 to cysteine cathepsins was evaluated in vitro by binding to RAW 264.7 cell lysates. VGT-309 bound cathepsins B, L, X, and S in a dose-dependent manner (Fig. 1c). Cathepsin binding to VGT-309 was abolished when cells were pre-incubated with the cysteine cathepsin inhibitor E64d.

### VGT-309 demonstrates tumor-specific labeling in 4T1 tumor allografts

**Fig. 2:**
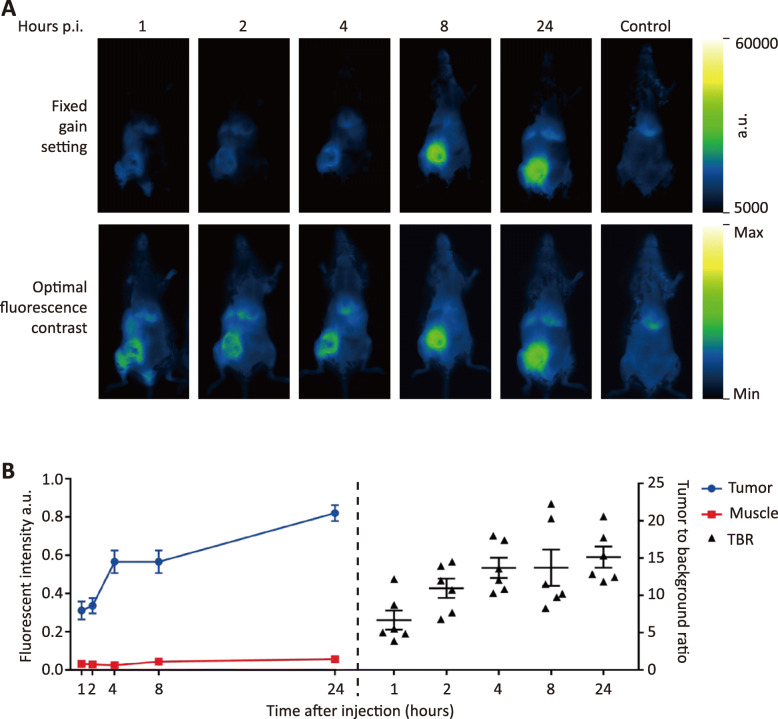
In vivo and ex vivo tumor fluorescent signal increases over time after 20 nmol VGT-309 injection. **a** Representative fluorescent images from the IVIS spectrum of tumor-bearing mice at different time points post intravenous injection VGT-309 and the control mouse without tumor inoculation. Six mice were evaluated per group. The upper panels demonstrate fluorescent images with the same scale. The lower panels show the optimal fluorescence contrast at each time point. **b** Quantitative fluorescent signal from the Pearl imager shows changes in fluorescent signal of tumor and background muscle tissue and tumor-to-background ratio (TBR) at different time point after intravenous injection of VGT-309. *n* = 6 for each time point. All data are presented as mean ± standard error

**Fig. 3:**
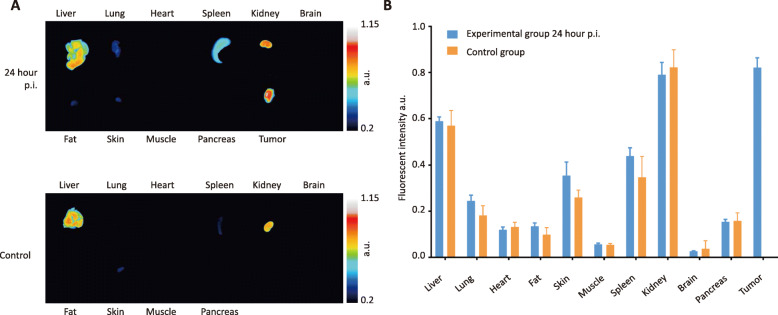
Comparable pattern in VGT-309 organ biodistribution between the experimental mice (24 h post injection group) and the control mice. **a** Representative fluorescent images of tissues from the experimental mice (upper panel) and the control mice (lower panel). **b** Quantitative fluorescent signal of **a**. *n* = 6. Data are presented as mean ± standard error. An independent Student’s *T* test did not find significant differences between groups

To preserve all sources of cathepsin proteases, including macrophages, a 4T1 mammary tumor allograft mouse model was chosen. Using an optimal fluorescence contrast setting, the tumor margin could be delineated by the fluorescent signal as soon as 1 h after injection of 20 nmol VGT-309. In vivo tumor fluorescent signals continued to increase up to 24 h after VGT-309 injection as demonstrated by using a fixed gain setting (Fig. 2a). Ex vivo organ biodistribution analysis confirmed that the fluorescent signal of tumors increased over time up to 24 h after VGT-309 injection (Fig. 2b, Supplementary Fig. S2). In contrast, the fluorescent signal of the background muscle tissues remained stable at different time points post probe injection (Fig. 2b, Supplementary Fig. S2). This resulted in an increased median tumor-to-background ratio, which was as high as 15.1 in the group evaluated at 24 h after probe injection (Fig. 2b). Based on these results, 24 h post probe injection was selected as the time point for imaging in the control group. In contrast to the experimental groups, fluorescent signal was not detected in the lower mammary fat pad of mice in the control group, as compared with the experimental groups with tumor inoculated in that area (Fig. 2a). Ex vivo organ biodistribution analysis revealed comparable fluorescent signals in the same tissues between the experimental mice and the control mice, except for the tumor (Fig. 3a, b). The strong signal in the liver and kidneys was expected and consistent with the finding that these tissues express high cathepsin levels [18].

### VGT-309 fluorescent signal correlates with tumor-infiltrated macrophages

**Fig. 4:**
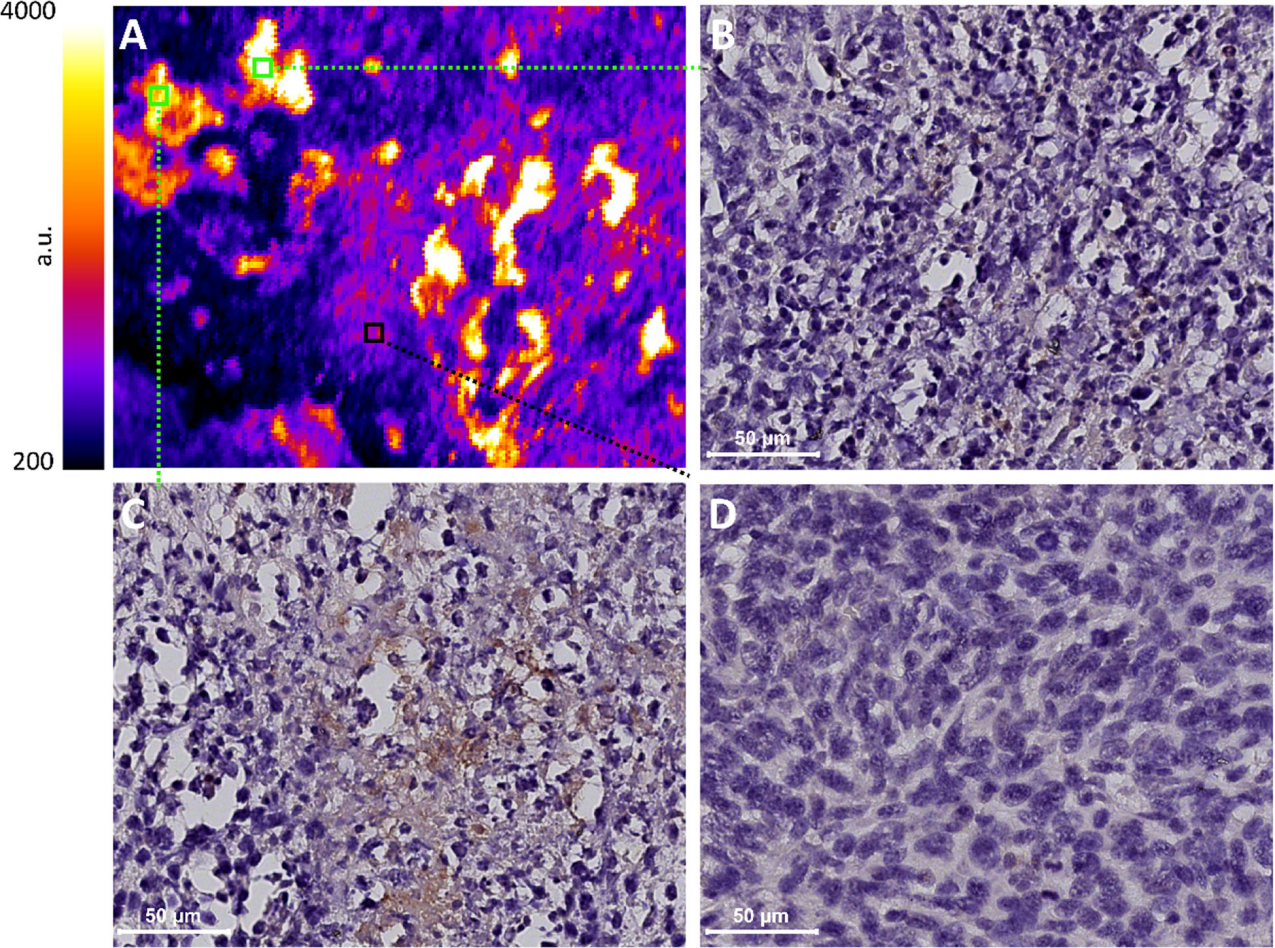
Microscopic fluorescent signal of VGT-309 colocalizes with tumor-infiltrated immune cells including macrophages. **a** A representative fluorescent image of a 4-μm-thick tumor slide from the mouse imaged 24 h after intravenous injection of VGT-309. Corresponding F4/80 immunohistochemical staining images of the areas highlighted by squares in **a** show **b** immune infiltrate containing few macrophages, **c** a high presence of macrophages, and **d** tumor cells

To determine the possible cellular source of VGT-309 fluorescent signal, we performed IHC staining using the murine macrophage marker F4/80. In the tumor tissue, the VGT-309 fluorescent signal was localized to the F4/80 positive staining area. Some immune cell enriched areas without F4/80 positive staining also demonstrated a high fluorescent signal (Fig. 4b), whereas VGT-309 signal could also be detected in the tumor area (Fig. 4d). In the spleen tissue, the fluorescent signal delineated the red pulp of the spleen, which also stained positive for F4/80 (Supplementary Fig. S3A). For muscle tissue, no F4/80 positive staining was found (Supplementary Fig. S3B).

### Image-guided surgery by intravenous injection of VGT-309 in a 4T1 tumor allograft model

**Fig. 5:**
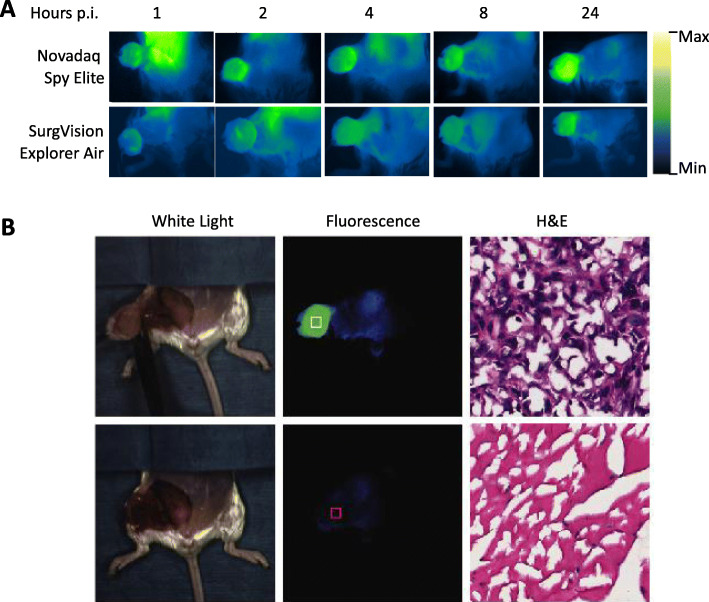
Image-guided surgery post intravenous injection of VGT-309. **a** Still images from Novadaq Spy Elite and SurgVision Explorer Air during resection of 4T1 tumors on different time points after injection of VGT-309. **b** White light (left) and fluorescent (middle) images of the surgical field before (upper) and after (lower) tumor removal using the Novadaq Spy Elite. H&E staining (right) histopathological analysis confirms that the tissues highlighted in the white and red squares in the middle panels are breast tumor and muscle, respectively

We further explored the potential utility of cathepsin-targeted imaging using VGT-309 for guiding surgical resection of the 4T1 tumors. Fluorescent images from each time point show clear tumor delineation after 2 h using the Novadaq Spy Elite and delineation at every time point with the Surgvision Explorer Air (Fig. 5a).

Image-guided surgery of mice 24 h after probe injection is shown in Video S1 and S2. The tumors were well-delineated by the fluorescent signal before removal. After tumor removal, the fluorescent signal in the tumor bed decreased dramatically (Fig. 5b, Video S1 and S2). Minor spillover signal was seen in the surgical cavity stemming from expected high liver signal. Nevertheless, in clinical practice, spillover signal is unlikely to be an issue since the distance between breast and liver tissue in humans is larger than in a mouse.


**Additional file 2: Video S1.** Fluorescent image-guided surgery by the Novadaq Spy Elite system 24 hours after intravenous injection of VGT-309.


**Additional file 3: Video S2.** Fluorescent image-guided surgery by the SurgVision Explorer Air system 24 hours after intravenous injection of VGT-309.

A biopsy was taken from the tumor bed after tumor resection, which was confirmed to be muscle tissue by histopathological examination (Fig. 5b).

## Discussion

This study demonstrates that VGT-309 in combination with optical fluorescent imaging can guide the surgical resection of tumor in a breast tumor-bearing syngeneic mouse model.

Unlike conventional imaging techniques, which discriminate tumor from normal tissue based on morphological and/or architectural changes, tumor-targeted optical molecular imaging enables in vivo real-time tumor detection based on molecular alterations in tumors [19]. This technique was first introduced in 2011 for intraoperatively guiding the surgical resection of tumors in patients [20]. Several tumor-specific optical fluorescent probes have been developed and investigated in both preclinical and clinical studies for tumor detection and fluorescent image-guided surgery [21–29]. Results from early-phase clinical trials indicate that it is feasible to incorporate fluorescent image-guided surgery in standard of care surgical procedure, as fluorescent image-guided surgery is safe for human use and had limited interference with the standard of care procedure [23, 25, 27, 20]. Moreover, studies have demonstrated potential clinical value of this technique in terms of improving intraoperative detection of tumor deposits or tumor positive resection margins [27, 28]. However, many of these probes are antibody-based with an optimal imaging time point several days after intravenous injection due to their relatively slow distribution. Therefore, patients need to have another visit to the hospital before surgery. Rapidly distributing probes that enable imaging several hours or one day after intravenous injection are desirable for clinical practice. Our study demonstrated that as soon as 1 h after VGT-309 injection, the tumor could already be detected by the fluorescent signal with the IVIS spectrum. Ex vivo, the tumor-to-background ratio increased from 6.7 at 1 h to 15.1 at 24 h after probe injection. Furthermore, the high tumor-to-background ratio clearly differentiated tumor from normal tissue enabling image-guided surgery already 2 h post VGT-309 administration with both clinical imaging devices. VGT-309’s flexible imaging window of 2 to at least 24 h after injection makes it a desirable probe for clinical practice.

To date, several biomarkers served as the targets in the development of tumor-specific probes for tumor detection in breast cancer. These markers include vascular endothelial growth factor A (VEGFA) [30], human epidermal growth factor receptor 2 (HER2) [31], gastrin-releasing peptide receptor (GRPR), and integrin α_v_β_3_ [32]. Probes targeting these biomarkers provided high tumor-to-background ratio for tumor detection or guiding surgical resection of tumors in preclinical and early-phase clinical trials [21, 28, 32]. Limitations for these biomarkers are, for example, that HER2 is overexpressed in 20–30% [33], and GRPR in around 76% [34] of breast cancer tumors. This limits the application of probes targeting these markers in all breast cancer patients.

Moreover, probes targeting VEGFA, GRPR, and α_v_β_3_ showed positive signal in normal tissue and false-negative tumor detection in early-phase clinical trials [28, 32]. These results leave room for improvement for breast tumor detection in the field of tumor-specific molecular imaging. Literature data showed that cathepsins, in particular cathepsin B, L, C, and S, are overexpressed in human primary breast cancer tissue and are associated with worse patient prognosis [11]. Both tumor-associated macrophages and breast tumor cells express cathepsins [35]. Our study showed that the qABP VGT-309 specifically bound to cathepsins B, L, X, and S. In in vivo experiments, this probe accumulated in breast tumor tissue but not in surrounding normal tissue. As expected, VGT-309 activation was also observed in the liver, spleen, and kidney tissues in the present study. This is probably due to cathepsin expression by resident macrophages in the liver and spleen, or the clearance of activated VGT-309 through the kidney, which may affect the application of VGT-309 for tumor detection in these organs. In line with literature, at the microscopic level we also found VGT-309 accumulation in macrophage-enriched areas and in areas with viable tumor cells. In human breast cancer, the majority of tumor-associated macrophages or tumor-infiltrating lymphocytes locate at the invasive front (or “margin”) of the tumors [36, 37]. Together, these data support the further evaluation of our probe VGT-309 on fluorescent image-guided intraoperative surgical margin assessment in breast cancer.

Cathepsin-targeting probes have been investigated for use in tumor detection and fluorescence-guided tumor surgical resection in preclinical studies or early-phase clinical trials [9, 10, 16, 38–44]. These probes are based on fluorescently labeled inhibitors or substrates. Substrate-based probes that do not include a tumor retention strategy diffuse rapidly from their substrate, resulting in fast clearance of the fluorescent signal from the target location. Moreover, substrate-based probes do not allow identification of the proteases which activate the probes. This makes it difficult to assign signal activated function to the probe-specific targets. For most of the reported activity (inhibitor)-based probes, far-red fluorophores are used for visualization of the probe-targeting cathepsins [9, 10, 16, 40, 41, 43]. Although promising tumor detection has been shown in preclinical mouse models with far-red activity-based probes, the low tissue penetration depth may prevent their use in in vivo human cancer detection. Moreover, these far-red fluorophores have not yet been approved for human use by FDA or EMA. In this study, we overcome these hurdles by using a FDA and EMA approved near-infra red fluorophore ICG for target visualization, which has a tissue penetration depth up to 10 mm and can be viewed on widely available surgical imaging systems. This makes the likelihood of clinical adoption of VGT-309 much higher compared to non-ICG and far red fluorophores.

## Conclusion

Due to the lack of an internationally standardized protocol for calibration of optical imaging devices, fluorescent intensity of the same probe obtained from different imaging devices can be dramatically different. In this study, we were able to use VGT-309 for guiding surgical resection of the implanted tumors with two different imaging devices. This result supports future investigation on the value of VGT-309 for fluorescent image-guided surgery in patients with breast cancer. Since cathepsins are also overexpressed in colorectal cancer, brain cancer, lung cancer, ovarian cancer, and soft tissue sarcoma [11, 45], VGT-309 can be considered for tumor delineation and resection in many different types of cancers. Clinical translation of VGT-309 is currently in progress.

## Supplementary information

**Additional file 1:.** Figure S1. Structure of BMV109 and VGT-309. Figure S2. Tumor fluorescent signal increases over time after VGT-309 injection. Quantified fluorescent images of organs of interest from all the experimental mice in different time points (1, 2, 4, 8 and 24 hours post VGT-309 injection). Figure S3. (A) Top left, a representative fluorescent image of a 4-μm thick slide of the spleen from the mouse imaged 24 hours after intravenous injection of VGT-309. Top right, corresponding F4/80 immunohistochemical staining images of the same tissue slide. Bottom, areas highlighted by squares in top left. (B) Left, H&E staining of muscle tissue imaged 24 hours after intravenous injection of VGT-309. Right, F4/80 immunohistochemical staining of the same muscle tissue.

## Data Availability

The datasets used and analyzed during the current study are available from the corresponding author on reasonable request.

## Abbreviations

EMA: European Medicines Agency
FDA: Food and Drug Administration
GRPR: Gastrin-releasing peptide receptor
H&E: Hematoxylin and eosin
HER2: Human epidermal growth receptor 2
ICG: Indocyanine green
IGS: Image-guided surgery
IHC: Immunohistochemical
PBS: Phosphate-buffered saline
PFA: Paraformaldehyde
qABP: Quenched fluorescent activity-based probe
ROI: Region of interest
