# Supplementary material for: Fluorescent image-guided surgery in breast cancer by intravenous application of a quenched fluorescence activity-based probe for cysteine cathepsins in a syngeneic mouse model
Source: EJNMMI Res. 2020 Sep 29;10:111. doi: 10.1186/s13550-020-00688-0 (PMC7524956; doi:10.1186/s13550-020-00688-0)
Supplement: Supplementary file 3 — Additional file 1:. Figure S1. Structure of BMV109 and VGT-309. Figure S2. Tumor fluorescent signal increases over time after VGT-309 injection. Quantified fluorescent images of organs of interest from all the experimental mice in different time points (1, 2, 4, 8 and 24 hours post VGT-309 injection). Figure S3. (A) Top left, a representative fluorescent image of a 4-μm thick slide of the spleen from the mouse imaged 24 hours after intravenous injection of VGT-309. Top right, corresponding F4/80 immunohistochemical staining images of the same tissue slide. Bottom, areas highlighted by squares in top left. (B) Left, H&E staining of muscle tissue imaged 24 hours after intravenous injection of VGT-309. Right, F4/80 immunohistochemical staining of the same muscle tissue. [file 13550_2020_688_MOESM1_ESM.docx]

**Supplemental data for**

**Fluorescent image-guided surgery in breast cancer by intravenous application of a quenched fluorescence probe for cysteine cathepsins in a syngeneic mouse model**

Frans V. Suurs^1†^, Si-Qi Qiu^1,2^*^†^, Joshua J. Yim^3^, Carolien P. Schröder^1^, Hetty Timmer-Bosscha^1^, Eric S. Bensen^6^, John T. Santini, Jr.^6^, Elisabeth G.E. de Vries^1^, Matthew Bogyo^3, 4, 5^, Gooitzen M. van Dam^5,6^*

**Supplementary figures**


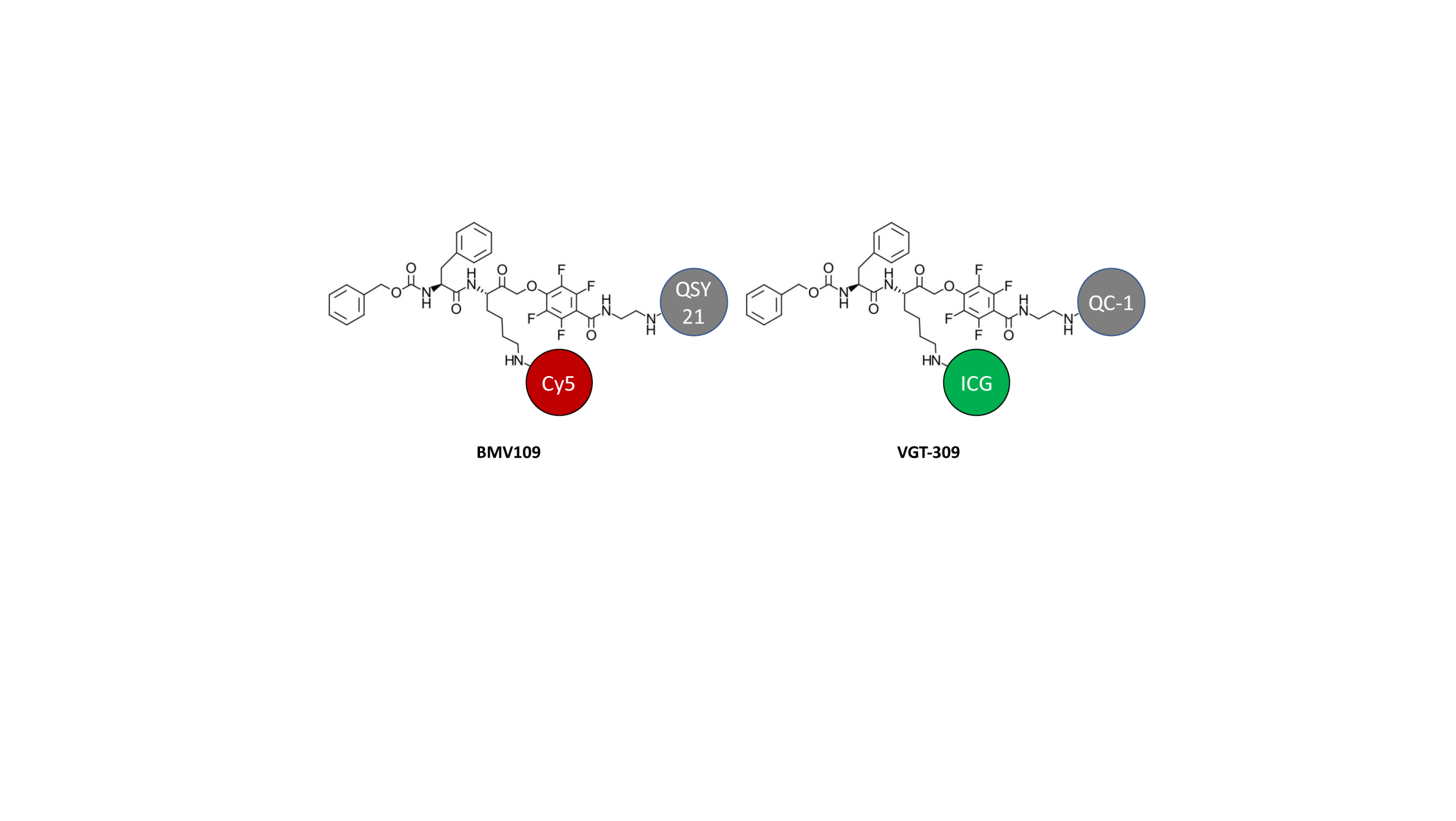


**Figure S1.** Structure of BMV109 and VGT-309.

**Figure S2.** Tumor fluorescent signal increases over time after VGT-309 injection. Quantified fluorescent images of organs of interest from all the experimental mice in different time points (1, 2, 4, 8 and 24 hours post VGT-309 injection).


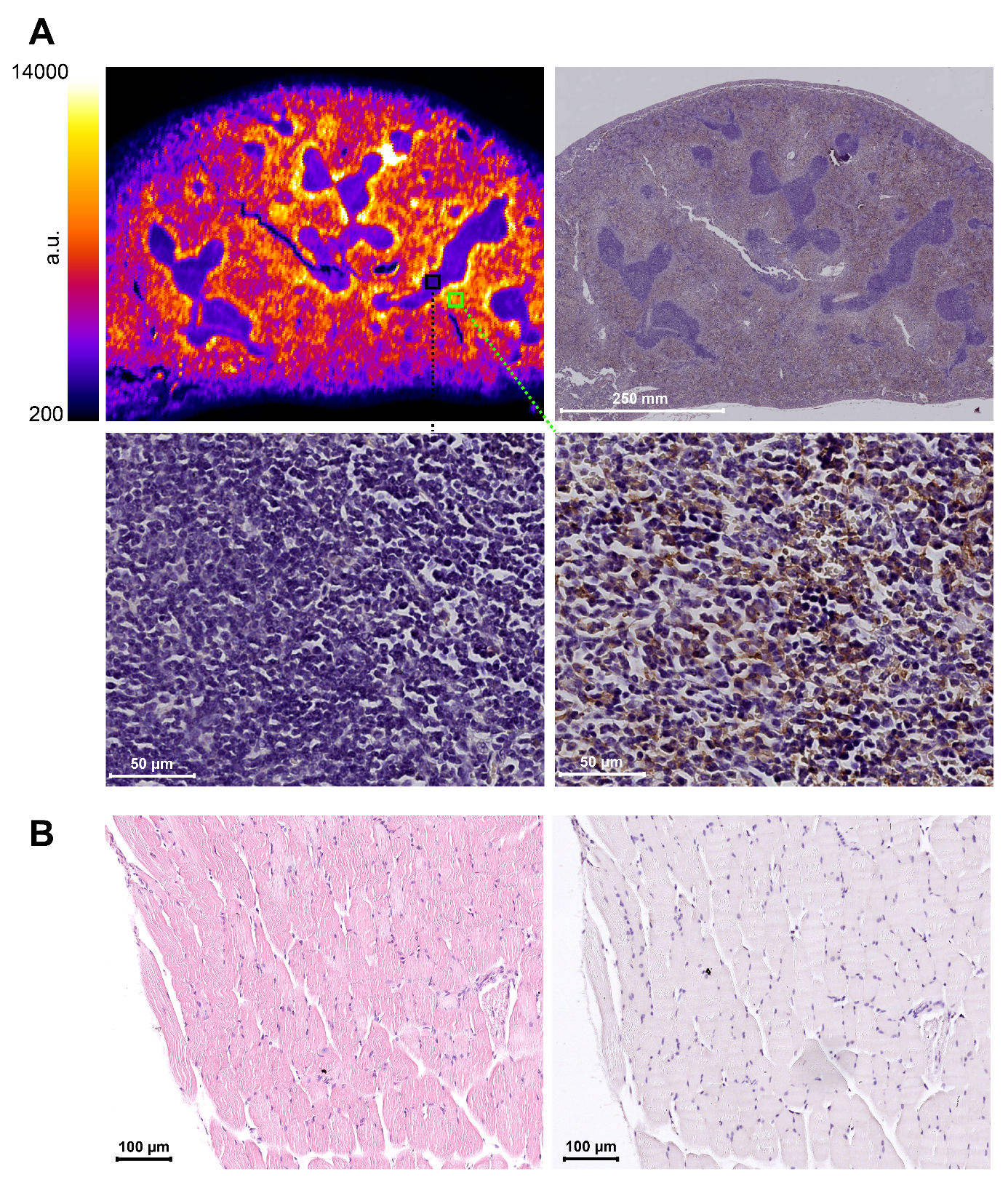


**Figure S3.** (A) Top left, a representative fluorescent image of a 4-µm thick slide of the spleen from the mouse imaged 24 hours after intravenous injection of VGT-309. Top right, corresponding F4/80 immunohistochemical staining images of the same tissue slide. Bottom, areas highlighted by squares in top left. (B) Left, H&E staining of muscle tissue imaged 24 hours after intravenous injection of VGT-309. Right, F4/80 immunohistochemical staining of the same muscle tissue.
